# Development of *Salmonella* Enteritidis vaccine candidate based on streptomycin independent suppressor and metabolic drift rifampicin resistance-attenuating markers

**DOI:** 10.1016/j.heliyon.2020.e04810

**Published:** 2020-08-28

**Authors:** Awad A. Shehata, Reda Tarabees, Mohamed Elsayed, Gamal Wareth, Shereen Basiouni

**Affiliations:** a Avian and Rabbit Diseases Department, Faculty of Veterinary Medicine, University of Sadat City, 32897, Sadat City, Egypt; b Research and Development Section, PerNaturam GmbH, Gödenroth, Germany; c Department of Bacteriology, Mycology and Immunology, Faculty of Veterinary Medicine, University of Sadat City, 32897, Sadat City, Egypt; d Friedrich-Loeffler-Institut, The Institute of Bacterial Infections and Zoonoses, 07743, Jena, Germany; e Faculty of Veterinary Medicine, Benha University, Moshtohor, 13736, Toukh, Egypt; f Department of Cardiothoracic Surgery, University Regensburg, Regensburg, Germany

**Keywords:** Microbiology, Infectious disease, Immunology, *Salmonella*, Streptomycin-independent, Metabolic drift, Vaccine

## Abstract

*Salmonella* is one of the most frequent food-borne pathogens and remains public health threat globally. The control of *Salmonella* in poultry, the main reservoir of non-typhoidal salmonellae, is a fundamental approach to ensure the safety of poultry products for human consumption. In the present study, a new live attenuated *Salmonella* enterica serovar Enteritidis vaccine candidate containing three attenuating markers based on streptomycin-independent (Sm-id) suppressor, and metabolic drift antibiotic resistance (MD- “res”) was developed. The streptomycin dependent (Smd) mutants were derived from *Salmonella* Enteritidis wild-type strain using streptomycin. Then the Sm-id mutants were derived from the isolated Smd mutants and designated “Smd→Sm-id”. A third MD- “res” marker was generated from Smd→Sm-id using rifampicin (Rif) and designated “Smd→Sm-id→Rif”. The colony sizes of these mutants were stable after more than 50 serial passages on blood agar; reversion to virulence can be almost excluded. The safety and efficacy of Smd→Sm-id and Smd→Sm-id→Rif were evaluated in one-day-old commercial layer chicks. Both mutants proved to be safe in terms of clinical signs, mortalities, lesion scores of visceral organs and rapid clearance when administered orally at a dose of 10^8^ colony forming unit (CFU), whereas birds inoculated with 10^8^ CFU *Salmonella* Enteritidis wild-type strain showed diarrhea, mortalities (3/40) and necrosis in liver and spleen. Chickens vaccinated with the developed mutants showed no seroconversion; however, wild-type strain induced a significant seroconversion at 3-week-postvaccination (wpv). The developed mutants protected chickens against challenge with 10^8^ CFU of *Salmonella* Enteritidis wild-type strain at 3-wpv. Vaccinated birds showed neither clinical signs nor mortalities during two-week post-challenge. In addition, the challenge strain could not be detected in pooled liver and spleen samples (0/5) at 7^th^ day post-inoculation (dpi). However, non-vaccinated challenged birds showed diarrhea and the challenge strain was re-isolated from pooled liver and spleen samples (3/5) at 7^th^ dpi. In conclusion, the developed mutants are safe and fully protected immunized chickens following heterologous challenge. It is obvious that the genetic characterization of these mutants and evaluation of different vaccination regimes are still in demand.

## 1 Introduction

Salmonellosis is one of the most important infectious diseases in humans and animals, as well as in food production sectors. *Salmonella* infections were responsible for about 80–93.2 million cases of foodborne illnesses worldwide (Crump et al., 2004; Majowicz et al., 2010). This number may be likely to increase as the global demand for poultry and poultry products increases, which necessitate the urgent need for strict surveillance-and-intervention strategies to reduce the contamination of such products with salmonellae, especially those of public health importance. Up to date, the genus *Salmonella* encompasses two species, six subspecies and includes over 2,600 serotypes based on the antigenic and biochemical characteristics (OIE, 2004; Issenhuth-Jeanjean et al., 2014; Gal-Mor et al., 2014). *Salmonella* enterica serovar Enteritidis infects chickens mainly via the fecal-oral route. It can colonize the intestine, invades visceral organs such as spleen and liver, and then spreads to the reproductive tract of the infected chickens (Chappell et al., 2009). *Salmonella* Enteritidis is considered one of the most critical zoonotic *Salmonella* commonly found in domestic poultry and responsible for many outbreaks in humans through the consumption of contaminated food, especially those prepared with raw eggs or other poultry products (Altekruse et al., 2006; Korsgaard et al., 2009; Stevens et al., 2009; Dorea et al., 2010; Antunes et al., 2016; Tarabees et al., 2017; Chousalkar et al., 2018).

Vaccination is more likely to have a fundamental role in the reduction of *Salmonella* infections in chickens (Van Immerseel et al., 2005; Shehata et al., 2013; Li et al., 2019; Redweik et al., 2020). Several inactivated *Salmonella* vaccines, including combined *Salmonella* Enteritidis and *Salmonella* Typhimurium vaccines, have been used for poultry. However, attenuated live *Salmonella* vaccines received more considerations as they can afford more protection by enhancing the production of the cell-mediated and mucosal immune responses (Gerdts et al., 2006). Previous reports have shown the ability of live *Salmonella* vaccines to stimulate the gut-associated lymphoid tissue (GLAT) and enhance the production of IgA (Mastroeni et al., 1999; Wahid et al., 2019). Live attenuated *Salmonella* Enteritidis vaccines induced protection in chickens (Guo et al., 2019; Li et al., 2019) and stimulated cell-mediated immunity such as IFN-γ, IL-1β, and IL-6 (Li et al., 2019).

Many live attenuated vaccines against *Salmonella* were developed and evaluated. The semi-rough strains dependent vaccines such as 9R and HWS51 have been produced and used against *Salmonella* Gallinarum and *Salmonella Dublin* infections, respectively (Mastroeni et al., 2001). Vaccines including *aro*A mutants and strains with mutations in the genes encoding adenylate cyclase and the cyclic adenosine monophosphate receptor protein have been developed based on molecular biological gene-deletion techniques and used as live vaccines against salmonellosis (Cooper et al., 1994). Additionally, attenuated vaccines include auxotrophic mutants or metabolic drift (MD) mutants vaccines against *Salmonella Enteritidis* have been also developed and used in Germany and in the United Kingdom (OIE, 2004). The MD is a function related mutation or spontaneous mutations of ribosomal RNA that occurs in all microorganisms (viruses, bacteria, yeast, and fungi) as an evolution principle.

In the present study, new live attenuated mutants containing a combination of Sm-id suppressors and MD resistance mutants against *Salmonella Enteritidis* were developed. In addition, the safety and efficacy of these mutants were evaluated in commercial layer chickens.

## 2 Materials and methods

### 2.1 Isolation and identification of *Salmonella* Enteritidis

*Salmonella* Enteritidis was isolated from one-week-old commercial layer chickens kept in the backyard. The diseased birds exhibited various clinical signs, including ruffling feathers and whitish diarrhea. For the initial isolation of *Salmonella* Enteritidis, the collected internal organs (pooled liver and spleen samples) were pre-enriched cultivated on Muller–Kauffman tetrathionate (Oxoid Ltd, Hampshire, England) at 37 °C for 24 h, followed by streaking on xylose lysine desoxycholate (XLD; Oxoid Ltd, Hampshire, England). The identification of suspected *Salmonella* colonies was conducted based on the biochemical tests, PCR, and Matrix-Assisted Laser Desorption/Ionization-Time of Flight (MALDI-TOF) as described previously (Shehata et al., 2013). The serotyping has been carried out using Anti-*Salmonella* A-E, A, B, C, D, and E (SIFIN, Berlin, Germany), Polyvalent (O) I, II, III and monovalent *Salmonella* O (Denka Seiken co., Japan) and H-antisera for both phase I and phase 2 (Denka Seiken co., Japan), according to manufacturers.

### 2.2 Determination of minimal inhibitory concentrations

Isolated *Salmonella* Enteritidis was passaged on Caso agar (3.5% Caso, 0.3% yeast extract, 0.1 glucose, 0.5% Agar Agar) (SIFIN, Berlin, Germany). The Minimal Inhibitory Concentrations (MIC) of streptomycin (Roth, Karlsruhe, Germany) and rifampicin (Rif) (Infecto Pharm, city, Germany) were determined in triplicate in a 96-well microtiter plate (Shehata et al., 2013). Briefly, 20 μl of *S*. Enteritidis [10^5^ colony forming unit (CFU)/ml] were added to 180 μl broth media containing different concentrations of streptomycin or rifampicin (1, 2, 4, 8, 16, 32, 64, and 128 μg/ml). Plates were then incubated overnight at 37 °C for 24 hrs. The antibiotic concentration that inhibited the growth of bacteria was regarded as a MIC value.

### 2.3 Vaccine candidate preparation

#### 2.3.1 Isolation of streptomycin dependent mutants (Smd)

The streptomycin dependent mutants “Smd” were isolated by culturing approximately 10^10^ CFU of fresh *Salmonella* Enteritidis wild-type strain on Caso agar supplemented with 1000 μg streptomycin per ml. Plates were incubated for at 37 °C for 48–72 h. A total of 500 raised colonies were sub-cultured on blood agar containing (1000 μg/ml) or free from streptomycin. Colonies that grow only on blood agar containing streptomycin were designated as “Smd” mutants.

#### 2.3.2 Isolation of streptomycin independent mutants (Sm-id) from Smd

The streptomycin independent mutants “Sm-id” were derived from Smd by culturing approximately 10^10^ CFU of fresh Smd on Caso agar free from antibiotics and incubated aerobically 37 °C for 48 h. Colonies grown on streptomycin -free blood agar and streptomycin -containing blood agar were considered as streptomycin independent (Sm-id mutants), and designated “Smd→Sm-id” mutants.

#### 2.3.3 Combination MD “Res” marker

Antibiotic-resistant mutants were generated by spatulating approximately 10^10^ CFU of fresh “Smd→Sm-id” mutants on Caso agar (SIFIN, Berlin, Germany) supplemented with 300 μg Rif per ml and incubated aerobically at 37 °C for 24 h. Small resistant colonies were passaged once on the antibiotic supplemented Caso ager then on antibiotic-free Caso agar. Stable diminished colony sizes were determined after 50 passages on Caso agar (SIFIN, Berlin, Germany), and these criteria served as a principle of stability and considered as Smd→Sm-id→Rif mutants.

### 2.4 Preparation of vaccine and challenge strains

The developed *Salmonella* Enteritidis mutants (Smd→Sm-id and Smd→Sm-id→Rif) and the wild-type strain were cultured on antibiotic-free Caso agar media (SIFIN, Berlin, Germany) and incubated aerobically at 37 °C for 24 h. Bacteria grown on each plate were harvested with dH_2_O, centrifuged at 3000 rpm for 5 min, and then washed twice with dH_2_O. The pellet was re-suspended in PBS, and the bacterial count was determined on Caso agar and expressed as CFU/ml.

### 2.5 Evaluation of safety and efficacy

All procedures used in the animal experiments were approved by the Animal Ethics Committee of Faculty of Veterinary Medicine, University of Sadat City, and complied with the Guidelines for the Care and Use of Animals in Research.

#### 2.5.1 Chickens

A total of 180 one-day-old commercial layer (Lohmann) chicks were used for evaluation of safety and efficacy of the developed mutants. *Salmonella*-free status was confirmed by bacteriological examination of meconium, obtained by abdominal squeezing, immediately after hatching (Data not shown). Chicks were housed under strict biosecurity conditions in five separate rooms located at the same facility, [G1 (n = 40), G2 (n = 40), G3 (n = 40), G4 (n = 30) and-G5 (n = 30)], Table 1. The room temperature was adjusted according to the age of the chickens following the recommendation for this genetic line. Birds were fed antibiotic- and anticoccidial-free balanced ration and free from meat or fishmeal. Feed and water were administered ad libitum. Feed and water were proven to be free from *Salmonella* using the culture method (data not shown).

**Table 1:** Experimental design for evaluation of safety and protective efficacy of MD-mutants in commercial layer chickens.

Group No.	no. of birds	vaccine/infection regime			challenge^a^		assessment of safety and efficacy
		Type	age/days	dose/bird (CFU)	Age/day	dose/bird (CFU)	
G1	40	smd→ sm-id^b^	1	10^8^	21	10^8^	1. Gross lesion scores^d^ 2. Clinical Signs 3. Mortality 4. Bacterial re-isolation^e^ 5. Seroconversion^f^
G2	40	smd→ sm-id→Rif^c^	1	10^8^	21	10^8^	
G3	40	*Salmonella* Enteritidis Wild-type strain	1	10^8^	-	-	
G4	30	-	-	-	21	10^8^	
G5	30	-	-		-	-	

a Challenge was done with 10^8^colony forming units (CFU) of *Salmonella Enteritidis* wild-type strain.

b “Smd→Sm-id” = Streptomycin independent (mutant containing two attenuating marker streptomycin (Smd) and streptomycin independent (Sm-id)).

c “Smd→Sm-id→Rif” = mutant containing three attenuating marker Smd, Sm-id and rifampicin resistant.

d Gross lesion score was calculated according to Nandre et al. (2015), whereas, “0” = no lesions, “1” = necrotic foci, “2” = enlarged and necrotic organs, “3” = more debilitated, necrotic, and distorted organ.

e Bacterial re-isolation of “Smd→Sm-id” and “Smd→Sm-id→Rif” was don on media containing 1000 μg streptomycin/ml, while *Salmonella* Enteritidis wild-type strain was done on antibiotic-free media.

f Seroconversion was done using ELISA at 3- weeks post vaccination or inoculation according to Shehata et al. (2013).

#### 2.5.2 Safety of MD-mutant vaccines

Birds kept in G1 and G2 were vaccinated orally using a sterile pipette at one-day-old with 100 μl PBS containing 10^8^ CFU of “Smd→Sm-id” or “Smd → Sm-id →Rif”, respectively. Chicks kept in G3 were inoculated orally with 100 μl PBS containing 10^8^ CFU *S*. Enteritidis wild type strain, while, chicks kept in G4 and G5 were not treated at one-day-old. The safety of these mutants was assessed based on clinical signs, mortalities, and invasiveness. At 3^rd^, 5^th^ and 14^th^ days post-inoculation (dpi), five chicks from each group were slaughtered and examined for the presence of necrotic foci on the livers and spleens. Re-isolation of *Salmonella* from pooled liver and spleen samples was carried out from slaughtered birds on Caso-agar (SIFIN, Berlin, Germany). Samples were considered negative when it still negative after enrichment in Selenite broth (Roth, city, Germany) for 16 h.

#### 2.5.3 Protection efficacy against a virulent challenge

Three-weeks post-vaccination, fifteen birds from G1, G2, and G4 were challenged orally with 10^8^ CFU of *Salmonella* Enteritidis wild-type strain (homologous strain) and kept under observation for further 15 days. Chickens kept in G5 served as negative control (non-vaccinated non-challenged) Protection was assessed based on clinical signs, mortality, and recovery of challenge strain from the internal organs. Five chickens per group were humanely euthanized, necropsied at 7^th^ and 14^th^ days post-challenge for bacterial recovery and post-mortem examination. Re-isolation of the wild-type strain from pooled liver and spleen samples (one gram from each) was carried out from slaughtered birds on Caso-agar containing antibiotics that used as a selectable marker (1000 μg streptomycin/ml). Samples were considered showing no growth after enrichment in Selenite broth (Roth, city, Germany) for 16 h. The mean lesion scores were carried out according to the methods described by Nandre et al. (2015). Individual gross lesions in the liver and spleen were given scores of 0–3 (“0” = no lesions; “1” = necrotic foci, “2” = enlarged and necrotic organs and “3” = more debilitated, necrotic, and distorted organs).

#### 2.5.4 Seroconversion

Blood samples (N = 5) were collected at 3-week-old from G1, G2, G3 and G5 and analysed using ELISA according to the previously developed protocol (Shehata et al., 2013).

### 2.6 Statistical analysis

All data are expressed as means ± standard deviations unless otherwise specified. The statistical analysis was carried out with GraphPad Prism 4 (Graph-Pad Software, La Jolla, USA). Two-way analysis of variance followed by unpaired Students *t*-test was used to identify significant differences between means.

## 3 Results

### 3.1 Isolation and identification of *Salmonella* Enteritidis

Suspected *Salmonella* colonies were identified by MALDI-TOF with score value > 2.300 and confirmed and further genotyped by PCR (data not shown). Based on serotyping, the isolated *Salmonella* was classified as *Salmonella* enterica var Enteritidis in which the “O” and “H” were {1,9,12} and {g,m}, respectively. The isolated *Salmonella* Enteritidis showed MIC values of 32 and <1.0 μg/ml for Streptomycin and Rifampicin, respectively.

### 3.2 Isolation of streptomycin dependent mutants

Many colonies with different sizes arose at 48 h after spatulating of fresh *Salmonella* Enteritidis wild type strain on Caso agar supplemented with 1000 μg of streptomycin per ml. About 500 colonies were passaged on streptomycin free- and streptomycin containing-blood agar media. Colonies which grown only in the presence of streptomycin are considered Smd. Due to the high MIC value of streptomycin (32 μg/ml), only five Smd colonies were obtained.

### 3.3 Isolation streptomycin independent mutants

**Figure 1:**
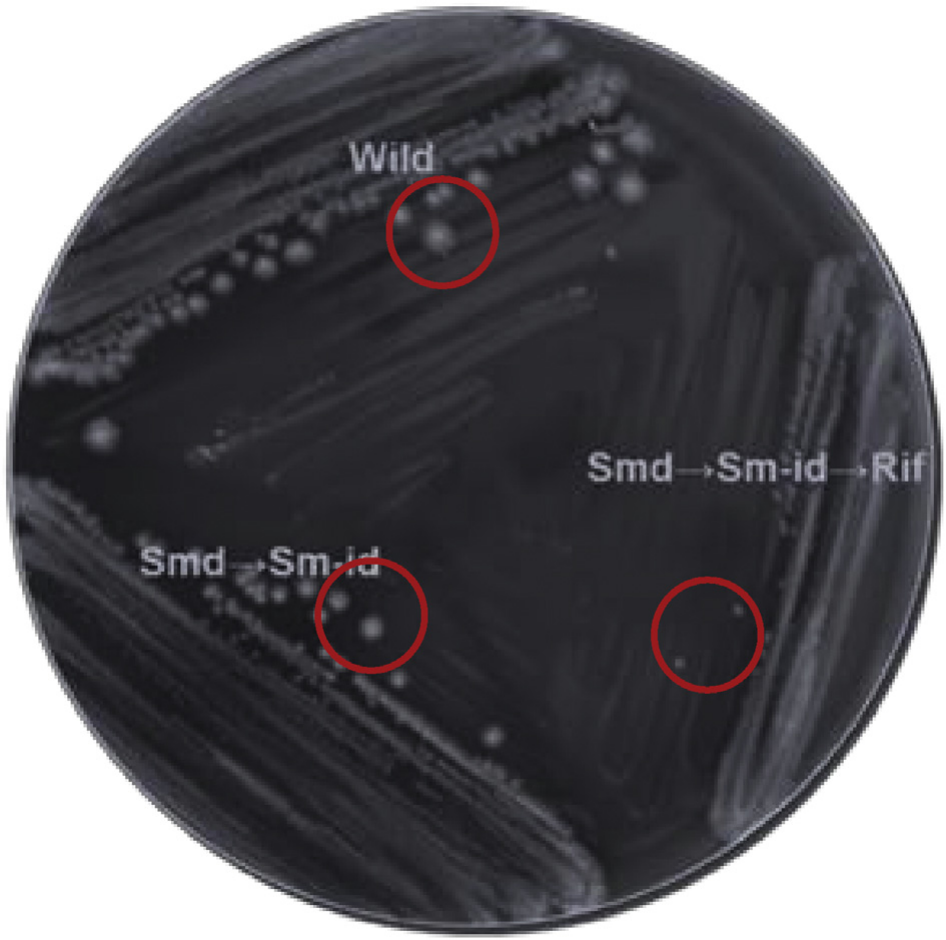
Colony morphology of *Salmonella* Enteritidis wild-type stain and metabolic drift-mutants showing gradually diminish the size. Streptomycin independent (“Smd→Sm-id”) mutants contains two attenuating marker streptomycin (“Smd”) and streptomycin independent (“Sm-id”). The “Smd→Sm-id→Rif” mutant contains three attenuating marker “Smd”, “Sm-id” and rifampicin resistant.

The Smd colonies were used for generating “Sm-id” mutants. Spatulating of 10^10^ CFU of fresh “Smd” on antibiotic-free Caso agar revealed different size colonies after 48–72 h. Many trials were done to get proper Sm-id colonies with a relative size around 70–80% of wild type (Figure 1). Only five suitable “Sm-id” were chosen from at least 400 colonies and one of them was selected randomly for the generation of the third attenuating marker.

### 3.4 Combination of MD “res” marker

The 3^rd^ marker was generated by spatulating of 10^10^ CFU of fresh “Sm-id” colonies on media containing Rif. Fifty colonies with different sizes were chosen and passaged on media containing Rif or free from antibiotics. Colonies that grown on the two types of media with a relative size of 30% of the wild type strains were considered as MD “res” and designated “Smd→Sm-id→Rif” (Figure 1).

### 3.5 Stability of the mutants

The probability of a back mutation of “Smd→Sm-id” and “Smd→Sm-id→Rif” can almost be excluded as the reduced colony sizes were stable after 50 passages on antibiotic-free blood agar media.

### 3.6 Safety of MD-mutant vaccines

**Figure 2:**
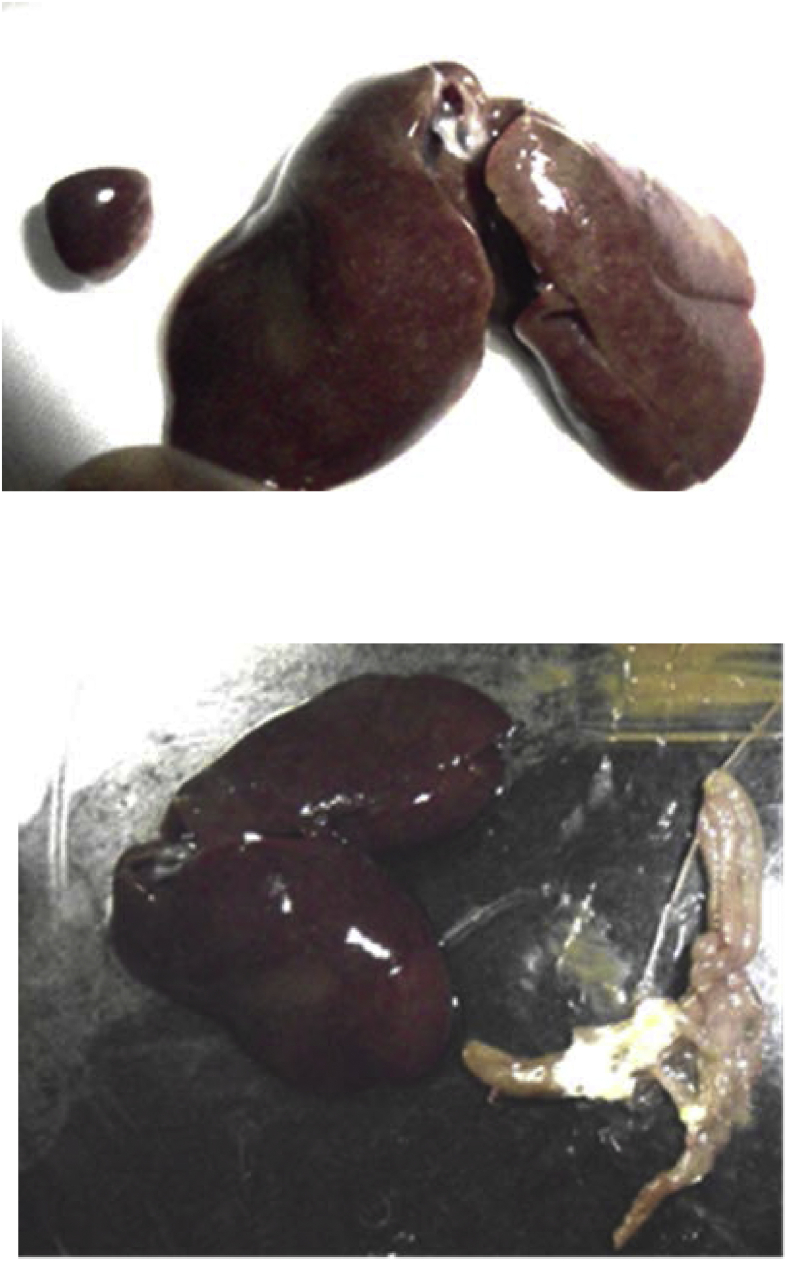
Chickens inoculated with 10^8^*Salmonella* Enteritidis wild-type strain at 3-week-old showed necrosis in the liver and spleen and whitish materials in the cecum at 7-day-post inoculation.

The “Smd→Sm-id” and “Smd→Sm-id→Rif” vaccines behaved as attenuated mutants in comparison with wild type strain in commercial layer chicks (Table 2). Chicks received Smd→Sm-id, or Smd→Sm-id→Rif1 mutants (10^8^ CFU) showed neither clinical signs nor mortalities during the three-week observation period. However, chicks inoculated with *Salmonella* Enteritidis (10^8^ CFU) wild-type at one-day-old showed diarrhea starting at 4^th^ dpi and mortality rate of 3/40. Postmortem examination at 7^th^ day post inoculation of chickens inoculated with *Salmonella* Enteritidis wild type (G4) showed necrosis in the liver and spleen and whitish materials in the cecum (Figure 2). *Salmonella* Enteritidis wild-type was re-isolated from pooled liver and spleen samples were (2/5) and (5/5) at 3^rd^ and 7^th^ dpi, respectively (Table 2). However, the “Smd→ Sm-id” and “Smd→ Sm-id→Rif” vaccines were re-isolated from pooled liver and spleen samples (3/5) only at 3^rd^ dpi. Although the developed mutants are invasive, they rapidly cleared from the body, and did not detect in liver and spleen at 7^th^ and 14^th^ dpi (Table 2).

**Table 2:** Safety of “Smd→Sm-id” and “Smd→Sm-id→Rif” –mutants in one-day-old commercial layer chickens.

Group. No.	No. of Birds	Mutants/Infection^a^	Safety		*Salmonella* re-isolation dpi^b^		
			Signs	Mortality^c^	3	7	14
G1	40	smd→ sm-id	-	0/30	3/5	0/5	0/5
G2	40	smd→ sm-id→Rif	-	0/30	3/5	0/5	0/5
G3	40	*S.* Enteritidis wild Strain	Diarrhea	3/40	5/5	2/5	0/5
G4	30	-	-	0/30	0/5	0/5	0/5
G5	30	-	-	0/30	0/5	0/5	0/5

a Vaccination and infection with 10^8^ CFU at a one-day-old orally.

b Spleen and liver samples collected from each bird were pooled for *Salmonella* re-Isolation at 3^rd^, 7^th^ and 14^th^ dpi.

c Mortality was started at 4^th^ dpi.

### 3.7 Protective efficacy against virulent challenge

The protective efficacy of smd→ sm-id and smd→ sm-id→Rif mutants was shown in Table 3. Following challenge with 10^8^ CFU, the non-vaccinated challenged (G4) birds had diarrhea while vaccinated birds (G1 and G2) showed no clinical signs. No mortalities were observed in all groups. *Salmonella* Enteritidis challenge strain was not detectable in the visceral organs of vaccinated chickens either at 7^th^ or 14^th^ dpi. However, it was detectable in pooled liver and spleen samples (3/5) at 7 dpi in non-vaccinated challenged birds. There were no gross lesions observed in the liver and spleen (lesion score = 0) of the vaccinated birds (G1 and G2). Non-vaccinated-non-challenged birds (G5) showed neither clinical signs nor mortality.

**Table 3:** Protective efficacy of “Smd→Sm-id” and “Smd→Sm-id→Rif” –mutants against heterologous challenge in commercial layer chickens.

Group No.	Vaccination^a^	No of Challenged birds^b^	Re-isolation of *Salmonella*^c^		Mean lesion scores ± SD^d^			
			7 dpi	14 dpi	Liver		Spleen	
					Enlargement	Necrotic foci	Enlargement	Necrotic foci
G1	Smd→ sm-id	15	0/5	0/5	0	0	0	0
G2	Smd→ sm-id→Rif	15	0/5	0/5	0	0	0	0
G4	Non-vaccinated-challenged	15	3/5	0/5	2.5 ± 1.2	2.3 ± 0.6	1.2 ± 0.7	1.4 ± 0.3
G5	Non-vaccinated-non challenged	-	0/5	0/5	0	0	0	0

a Vaccination with developed mutants was done at 1-day-old with 10^8^ CFU.

b Challenge was done orally with 10^8^ CFU *Salmonella* Enteritidis wild-type strain at 3^rd^ week post vaccination.

c Spleen and liver samples collected from each bird were pooled for *Salmonella* re-Isolation.

d Mean lesion scores ± Standard deviation at 7^th^ day post inoculation was calculated according to Nandre et al. (2015); whereas, 0 = no lesions, 1 = necrotic foci, 2 = enlarged and necrotic organs, 3 = more debilitated, necrotic, and distorted organ.

### 3.8 Humoral immune response

**Figure 3:**
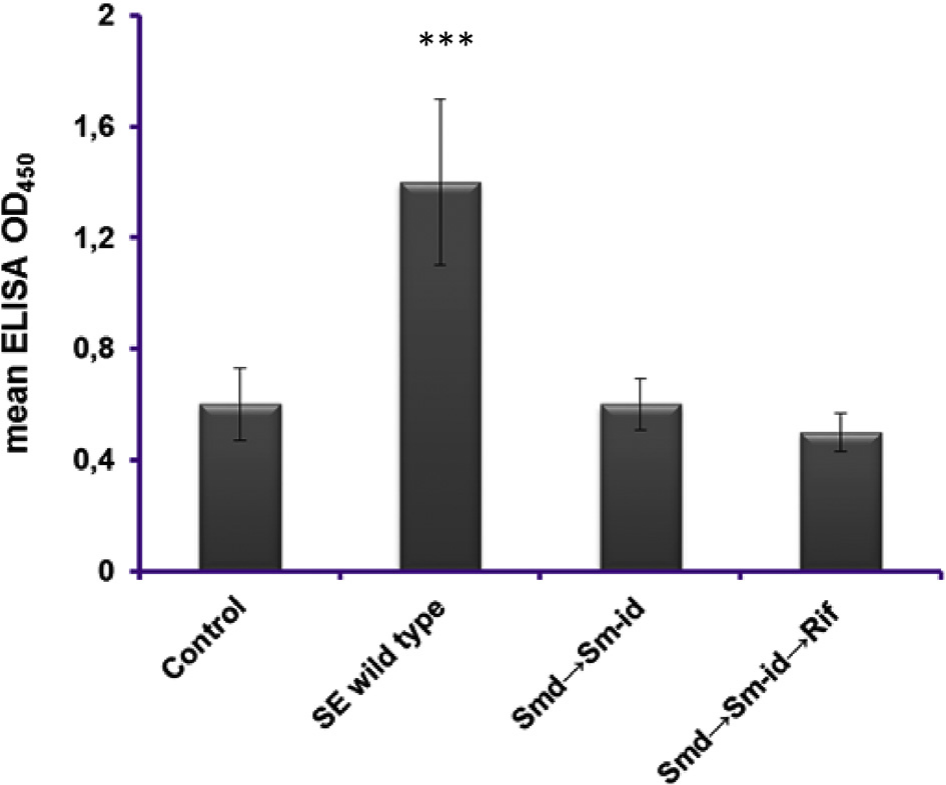
Mean Optical densities (OD_450_) of serum IgG in commercial layer chickens (n = 5) measured using ELISA following oral administration of different MD mutants at 3-weeks post inoculation. Both streptomycin independent (Smd→Sm-id) and streptomycin independent containing metabolic drift rifampicin as an additional attenuating marker (Smd→Sm-id→Rif) did not induce significant seroconversion compared with non-treated control chickens (G5) at 3-weeks post inoculation. Chickens inoculated with *Salmonella* Enteritidis wild type strain induced a significant seroconversion at 3 weeks post inoculation. Asterisks (∗∗∗) indicate significant increase (P < 0.0001) of antibody levels compared with negative control and mutants vaccinated chickens.

At 3^rd^ weeks post vaccination, the serum IgG levels were significantly higher (p < 0.0001) in the sera of challenged birds (G3) compared with those of the control (G5) and vaccinated groups (G1 and G2). Vaccinated birds showed no significant seroconversion compared with the non-treated broiler chickens (Figure 3).

## 4 Discussion

The primary aim of *Salmonella* control programs in large-scale poultry operations is to reduce the contaminations of various poultry products with *Salmonella*, especially that of public health importance and consequently decrease the human infection with these pathogens. Several live attenuated *Salmonella* vaccines were developed based on MD spontaneous mutation (Linde et al., 1995a, b, 1998; Mizuno et al., 2007; Revolledo and Ferreira, 2010; Shehata et al., 2013). This method was used also for development of live attenuated vaccines against other pathogens i.e., *Brucella melitensis* (ELBerg and Faunce, 1957), *Listeria* (Fensterbank, 1986), *Listeria monocytogenes* (Linde et al., 1995a, b), *Aeromonas hydrophila* (Pridgeon and Klesius, 2011a), *Streptococcus iniae* (Pridgeon and Klesius, 2011b), and *Edwardsiela ictaluri* (Pridgeon and Klesius, 2011c).

There are three methods for the development of MD mutants: 1) spontaneous MD antibiotic resistance (MD “res”) colonies which can be isolated with a frequency of 1% related to the virulent resistant colonies; 2) increased environmental stress tolerance (iet) mutants which indirectly accumulated in the “dying off” culture, and 3) streptomycin independent (Sm-id) suppressor mutants derived from streptomycin dependent (Smd) colonies (Shehata et al., 2013; Linde and Grosse-Herrenthey, 2015).

In the present study, live attenuated *Salmonella* Enteritidis vaccine candidate based on Sm-id suppressor and MD “res” attenuating markers was developed and evaluated in commercial layer chicks. The attenuation of bacteria using MD method induces spontaneous mutation of ribosomal RNA in which the incidence of back mutation per one attenuating marker may reach 10^−8^, in relation to the observed frequency of back mutations of spontaneously attenuated *Shigella*-tested in volunteers which was about 10^−8^ (Formal et al., 1971; Linde et al., 1995a, b; Linde and Grosse-Herrenthey, 2015). Different attenuation markers that increase the stability of vaccines are required to exclude the probability of a reversion to virulence. However, hyper-attenuated mutants could lose their immunogenicity, and this is a limitation of MD “res” containing multiple attenuating markers.

In 1985, Percy and co-workers developed Smd vaccine against *Pasteurella multocida.* This vaccine is a modified inactivated vaccine; that can multiply only in the presence of streptomycin. Although this vaccine protected against *Pasteurella multocida*, it was not used commercially due to safety concern, it contains only one attenuating marker (Percy et al., 1985). In the present study, mutants containing three attenuating markers based on “Sm-id” suppressors and MD “res” were developed. The incidence of back mutation of the developed mutants is estimated to be 1:10^24^, one chance each 100 million chances. The relative colony sizes of “Smd→Sm-id” and “Smd→Sm-id→Rif” to the *Salmonella* Enteritidis wild type were 70% and 30%, respectively (Figure 1). This reduction in the colony sizes could be attributed to functional mutations, which lead to increased generation time. Consequently, the virulence is reduced and can be used for vaccination purposes (Linde et al., 1995a, b). However, the gradually reduced colony sizes are inversely correlated with the degree of attenuation (Shehata et al., 2013; Linde and Grosse-Herrenthey, 2015).

Interestingly, the probability of a back mutation of the developed mutants can almost be excluded, as the reduced colony sizes were stable after 50 passages on blood agar media that meet the security demands of the World Health Organization (WHO). An additional advantage of these mutants is the ability to differentiate between the vaccinal and wild-type strains using differential culture media, which are easily achieved by the addition of the antibiotics involved in the genetic changes (Linde et al., 1995a, b; Shehata et al., 2013; Linde and Grosse-Herrenthey, 2015).

The attenuation of *Salmonella* Enteritidis (“Smd→Sm-id” and “Smd→Sm-id→Rif”) reduces pathogenicity in which neither clinical signs nor mortalities were observed in the vaccinated groups. However, the mutants retain immunogenicity, so it has the ability to invade internal organs such as liver and spleen. Both “Smd→Sm-id” and “Smd→Sm-id→Rif” mutants proved to be invasive but rapidly cleared from the body. This is probably because live strains can survive in the host for a short time and thereby mimic a natural infection (Griffin and Barrow, 1993; Shehata et al., 2013).

Previous studies have shown that oral administration of live *Salmonella* vaccines significantly stimulated the humoral and cell-mediated immune response and protected against infection with related *Salmonella* serovars compared with killed vaccines (Holt et al., 2003; Mohler et al., 2008; Matulova et al., 2013; Nandre et al., 2015; Eeckhaut et al., 2018). In the present study, birds orally vaccinated with “Smd→Sm-id” and “Smd→Sm-id→Rif” mutants showed no significant seroconversion, compared with non-vaccinated challenged birds. Similar results were obtained by *Salmonella* Gallinarum mutants (Shehata et al., 2013). Besides, some studies detected specific antibodies against *Salmonella Enteritidis* in sera of spray vaccinated young chickens but were not in sera of orally inoculated young chicks (Mizumoto et al., 2004; Adriaensen et al., 2007). It is postulated that *Salmonella* Enteritidis mutants stimulate the cellular or local immunity leading to the prevention of colonization of challenge bacteria, further studies are needed to evaluate the cellular immune response of the developed mutants. Hence, both cellular and humoral immunity are important in the defense against intracellular bacteria such as *Salmonella* Enteritidis. The combined use of attenuated and inactivated vaccines might provide a useful way to control *Salmonella* Enteritidis. Further studies will be conducted to evaluate different vaccination regimes.

## 5 Conclusion

Vaccination against non-typhoidal *Salmonella* is supreme to prevent the infection in poultry industry and consequently reduce risk in food chain and humans. The developed MD mutants (“Smd→Sm-id” and “Smd→Sm-id→Rif”) proved to be safe and protected chickens against oral challenge with homologous wild strain, providing effective vaccine candidate in terms of clinical signs, mortalities rates and pathological lesions. Oral application of such live attenuated vaccine candidate might reduce the burden of infection and could be a useful tool for the prevention of *Salmonella* in poultry, especially if combined with good biosecurity. However, further studies are needed for genetic characterization of the developed mutants and evaluation of some vaccination regimes in different poultry species. Evaluation of priming vaccination of commercial layers with MD mutants and boostering with the inactivated vaccine are in progress.

## Declarations

### Author contribution statement

Awad A. Shehata, Shereen Basiouni: Conceived and designed the experiments; Performed the experiments; Analyzed and interpreted the data; Wrote the paper.

Reda Tarabees, Mohamed Elsayed: Contributed reagents, materials, analysis tools or data; Wrote the paper.

Gamal Wareth: Contributed reagents, materials, analysis tools or data.

### Funding statement

This research did not receive any specific grant from funding agencies in the public, commercial, or not-for-profit sectors.

### Competing interest statement

The authors declare no conflict of interest.

### Additional information

No additional information is available for this paper.
